# P09 Does a virtual pain education workshop increase young person understanding of chronic pain, and confidence to self-manage?

**DOI:** 10.1093/rap/rkac067.009

**Published:** 2022-09-28

**Authors:** Aditi Surelia-Chauhan, Alexandra Orchard, Ellie Potts

**Affiliations:** Evelina London Children's Hospital, London, United Kingdom; Evelina London Children's Hospital, London, United Kingdom; Evelina London Children's Hospital, London, United Kingdom

## Abstract

**Introduction/Background:**

Chronic Pain is a complex condition. Our approach is to enable young people to function despite their pain. All patients are offered our Pain Education Workshop (PEW) based on pain education, self-management, and cognitive behavioural therapy. Pre-pandemic this was delivered face to face, and with social distancing guidelines in place we adapted virtually in response to patient need. We were interested to see if the children and young people were finding the intervention useful in their journey towards managing their pain. We hoped this would prove to a positive experience for our patients in a time of great uncertainty.

**Description/Method:**

Data was collected from patients who attended a weekday virtual PEW (a 3-hour multidisciplinary biopsychosocial intervention) at Evelina London Children’s Hospital between November 2020 and May 2022. All patient had an initial appointment in our multidisciplinary Paediatric and Adolescent Chronic Pain Clinic. Overall, the aims of the workshop are for patients to learn and recognize the link between psychosocial factors and the impact of boom and bust on pain intensity, whilst supporting patients’ ability to self-manage.

Attendees completed a series of pre and post workshop self-report questionnaires. The questionnaires requested patients to rate their confidence in their understanding of pain and in using self-management strategies, using a 5 point Likert scale (1=Not at All to 5=Extremely Confident). Patients were then asked an open question to establish what they would like to gain from the workshop. Immediately post workshop patients were asked to re-rate their confidence in their understanding of pain and in using self-management strategies. They were also asked to report what they will do differently as a result of attending the workshop.

A statistical comparison of the patients’ confidence in understanding chronic pain before and after PEW; and in self-management strategies were assessed with paired t-tests. Our null hypothesis being that no difference pre and post workshop would be found.

A qualitative content analysis of the patient’s reported hopes for (pre-workshop) and what they took away (post workshop) was undertaken and key themes were identified.

**Discussion/Results:**

Since November 2020, a total of 140 patient were offered PEW, 107 of which undertook this intervention. Group sizes varied from 4-14 young-person and parent diads, over 17 sessions. Full data sets for pre- and post-intervention feedback were analysed on 56/107 patients. These patients’ average age was 14 (range 9-18); 47 female, 9 male.

The mean patient self-reported understanding of chronic pain pre-workshop was 2.8/5 (range 1-5). Post-workshop scores increased to 4.0/5 (range 3-5). Patient’s confidence in managing their pain increased from 3.2/5 (range 2-5) to 3.9/5 (range 2-5). Paired T-tests on these scores showed a statistically significant difference in understanding chronic pain post PEW (P < 0.001), and in confidence in self-management post PEW (P < 0.001).

Themes identified pre-intervention by the young people included:

• Increasing understanding and knowledge of chronic pain

• learning how to cope with pain

• Improving access to treatment

Take home message themes:

• relaxation techniques

• pacing

• goal setting.

Analysis of the themes in combination with the patient reported scores demonstrated that outcomes as set by the young person were met by the intervention.

The results from this study provide evidence for the effectiveness of PEW, in terms of delivering predefined workshop outcomes. It showed significant improvements in patients understanding of chronic pain, and patient’s confidence in using self-management strategies. Similarly, when asked what they took away from the intervention, patients reported wanting to practice using self-management strategies-reinforcing the assumption that PEW was effective in increasing patients confidence to self-manage. As a result, the findings are supportive of assumptions that CBT interventions combined with pain education can have a positive impact, increasing patient’s self-efficacy and increasing their confidence to self-manage.

**Key learning points/Conclusion:**

In conclusion, this study provides evidence for the effectiveness of a brief multidisciplinary biopsychosocial intervention for chronic pain.

Previous research into the field has mainly focused on face-to-face interventions run over numerous weekly sessions. We have demonstrated similar effectiveness whilst using an online, one-off intervention. This demonstrates services can access patients and offer effective treatment whilst reducing the impact on resources. Moreover, we provided much needed virtual support for young people during and post pandemic.

Limitations:

• Small sample size. Larger sample is needed to fully assess the effectiveness of virtual PEW and to generalise to a wider population.

• High attrition rate (33/140). Paediatric patients with chronic conditions are more likely to show persistent/chronic absences (school attendance <90%). Research has shown that psychoeducational interventions have been most effective when they are run on weekends to minimise the loss of school. Access to technology to participate in a virtual group may also be a factor.

It is likely that further longitudinal studies are needed to assess the long-term effectiveness of PEW. We are unable to predict whether patients remained confident in using self-management strategies after the workshop or for how long. Since chronic pain is recurrent pain lasting longer than 3 months it is important to capture how effective PEW is in increasing patient’s confidence in managing their condition in the long-term. It is also necessary to assess the long-term effectiveness of PEW in increasing and improving patients understanding of pain. Studies have shown recall can improve 9-24 hours after new information is learnt. However, past 24 hours, recall of learnt information starts to decline if the information is not relearned continuously. Not being able to recall pain messages can affect patients’ abilities to utilise this knowledge to inform coping strategies and manage symptoms long term.

